# Serum Uric Acid-Lowering Effects of Combined Glycine and Tryptophan Treatments in Subjects with Mild Hyperuricemia: A Randomized, Double-Blind, Placebo-Controlled, Crossover Study

**DOI:** 10.3390/nu11030564

**Published:** 2019-03-06

**Authors:** Shunji Oshima, Sachie Shiiya, Yasunori Nakamura

**Affiliations:** Core Technology Laboratories, Asahi Group Holdings, Ltd., 1-21, Midori 1-Chome, Moriya-shi 302-0106, Japan; sachie.siiya@asahigroup-holdings.com (S.S.); yasunori.nakamura@asahigroup-holdings.com (Y.N.)

**Keywords:** glycine, tryptophan, uric acid, urinary pH, triglyceride

## Abstract

We determined the serum uric acid-lowering effects of combined daily supplementation of glycine and tryptophan in patients with mild hyperuricemia using a randomized, double-blind, placebo-controlled, crossover clinical trial design. Japanese healthy adult males and females with mild hyperuricemia (fasting serum uric acid of 6.6–7.9 mg/dL) ingested a powder mixture containing 3.0 g of glycine and 0.2 g of tryptophan or a placebo powder once daily at bedtime for 6 weeks. Combined supplementation with glycine and tryptophan significantly decreased serum uric acid levels (from 7.1 mg/dL to 6.7 mg/dL, *p* = 0.004) before and after the trial. Serum uric acid concentrations significantly decreased in the subjects supplemented with the amino acid mixture compared with those in placebo-treated subjects (*p* = 0.028). In addition, the combination treatment with glycine and tryptophan decreased serum triglyceride levels (from 119 mg/dL to 86 mg/dL, *p* = 0.002). Increased solubility of uric acid caused by urinary pH were likely contributors to the serum uric acid-lowering effects of the amino acid mixture.

## 1. Introduction

Hyperuricemia is an abnormal condition characterized by increased serum concentrations of uric acid, and it is the cause of gout [1]. It is defined as a condition in which the serum uric acid level exceeds 7.0 mg/dL, independently of sex and age [2]. Gout is a progressive metabolic disease characterized by symptomatic hyperuricemia and deposition of monosodium urate crystals in joints and soft tissues. The prevalence of gout has increased globally [3,4,5,6], and its progression indicates an imbalance of uric acid synthesis and excretion [7]. It has been suggested that hyperuricemia is in large part caused by decreased kidney excretion of uric acid [8], reflecting the relative contribution of renal uric acid excretion to the maintenance of serum uric acid levels. 

It is generally accepted that high protein diets increase the excretion of endogenous uric acid [9]. In previous studies, the non-essential amino acid glycine increased urinary excretion of uric acid in rats and in several healthy human males [10,11]. Similarly, following oral administration of glycine in 12 gout patients for three days, decreased plasma uric acid levels were observed in eight cases and increased urinary uric acid excretion was observed in nine cases [12]. These preliminary results suggest that continual ingestion of glycine effectively manages serum uric acid levels in hyperuricemic patients. Because little is known of the effects of other amino acids on uric acid excretion, we aimed to identify the amino acids that synergistically improve the effects of glycine on serum uric acid levels. In preliminary human studies, we observed transient declines in serum uric acid levels following oral administration of glycine with the essential amino acid tryptophan and found that the effect was optimal with comparatively smaller quantities of tryptophan (unpublished data). The present randomized, double-blind, placebo-controlled, crossover clinical trial was designed to confirm the serum uric acid-lowering effects of continual combined supplementation with glycine and tryptophan under conditions of mild hyperuricemia (serum uric acid levels of 6.6–7.9 mg/dL).

## 2. Materials and Methods

### 2.1. Participants

This intervention study was conducted in accordance with the Declaration of Helsinki, and the protocol was approved by the Ethics Committees of Nihonbashi Egawa Clinic (approval number: RAB16-004). Participants were recruited through the Internet in Japan. Written informed consent was obtained from all participating volunteers. Inclusion criteria for the study were as follows: Japanese healthy males and females aged 20–64 years with fasting serum uric acid levels of 6.6–7.9 mg/dL were recruited for the study and provided written informed consent prior to participating. Volunteers with a history of liver, renal, heart, or severe disease, diabetes, mental disorder, drug or alcohol dependence, drug or food allergy, or routine use of drug or dietary supplements for hyperuricemia, and those pregnant or lactating, were excluded from the study. A power calculation before the start of the study indicated that thirty subjects would have to be recruited to achieve a study power of 0.8 at a significance level of 0.05. Thirty-two of 178 subjects who received screening tests were enrolled according to the above criteria.

### 2.2. Test Compounds

Commercially available glycine, L-tryptophan, and dextrin were purchased from Yuki Gosei Kogyo Co. Ltd. (Tokyo, Japan), Ajinomoto Healthy Supply, Inc. (Tokyo, Japan) and Matsutani Chemical Industry Co., Ltd. (Tokyo, Japan), respectively. Dextrin was used as a placebo. All subjects ingested powdered test foods (active or placebo), which were made from the components listed in Table 1. Dextrin was added to the active powder to increase the fluidity. Active and placebo powders were supplemented with small amounts of lemon flavor and citric acid so that they could not be distinguished from each other. 

### 2.3. Study Design

**Figure 1:**
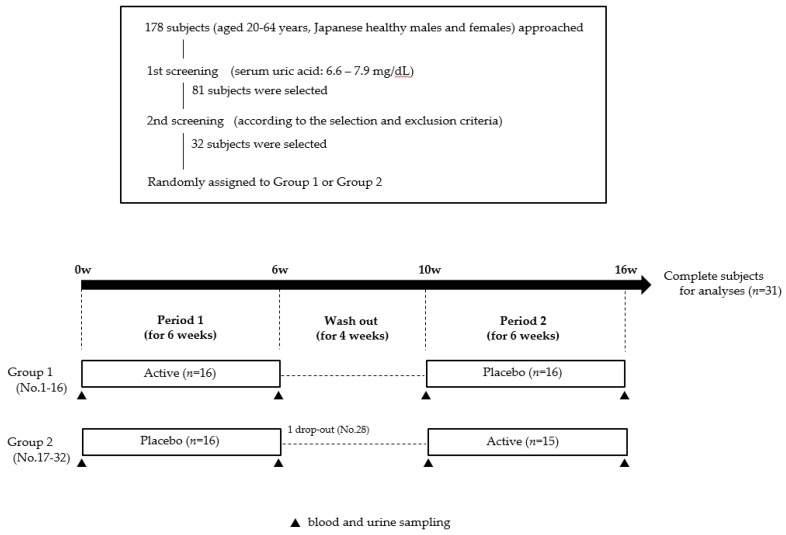
Experimental schedule showing the randomized, double-blind, placebo-controlled, crossover study design. The study was performed over a 16-week period.

The randomized, double-blind, placebo-controlled, crossover study design was registered with the University Hospital Medical Information Network (UMIN-CTR; registered ID: UMIN000033359, registration date: 30 July 2018) as shown in Figure 1. Thirty-two subjects were randomly allocated to Groups 1 or 2 using a block random sequence. Group 1 received placebo during period 1 and the active amino acid mixture during period 2 (placebo then active) and Group 2 received the reverse regimen (active then placebo). During the study period of 16 weeks (Periods 1 and Period 2 and the wash out period), all subjects were instructed to maintain daily eating and drinking habits and normal levels of daily physical activity. Subjects ingested mixed powders containing 3.0 g of glycine and 0.2 g of tryptophan or placebo powder once daily at bedtime for 6 weeks. Peripheral blood was collected from the cubital vein in the morning under overnight fasting conditions at the beginning and end of periods 1 and 2. Urine specimens were collected for 60 min after complete urine excretion following ingestion of 500 mL of water, and urine volumes were then recorded.

### 2.4. Blood and Urine Analyses

Serum uric acid, total protein, albumin, aspartic aminotransferase (AST), alanine aminotransferase (ALT), lactate dehydrogenase (LDH), gamma-glutamyl transferase (GGT), alkaline phosphatase (ALP), total bilirubin, creatinine, blood urea nitrogen (BUN), HDL-cholesterol (HDL-C), LDL-cholesterol (LDL-C), triglyceride (TG), blood glucose, and Hemoglobin A1c (HBA1c) levels, and white blood cell counts, red blood cell counts, hemoglobin, hematocrit, and platelet counts, urinary uric acid, creatinine, and potential of hydrogen (pH) were measured by a local clinical laboratory (LSI Medience Corporation, Tokyo, Japan). Urate and creatinine clearances were calculated using the following formula according to Du Bois et al. [13]: Urate or creatinine clearance (mL/min) = urinary urate or creatinine excretion (mg/min) / serum urate or creatinine concentration (mg/mL) × 1.73 (m^3^) / body surface area (m^3^). The normal range of urate clearance in healthy Japanese subjects is 6.2–12.6 mL/min [14]. Serum glycine and tryptophan concentrations were analyzed using an automated precolumn derivatization amino acid analytical method based on HPLC/electrospray ionization mass spectrometry (UF-Amino Station system; Shimadzu, Japan), as previously described [15]. 

### 2.5. Statistical Analysis

All statistical analyses were performed using BellCurve 2.15 software (SSRI, Tokyo, Japan). Serum uric acid levels were calculated as the primary outcome variable and were analyzed for carryover and period effects [16]. Temporal changes (before ingestion vs. 6 weeks later) in each variable were analyzed using paired *t*-tests and are presented as means ± standard deviations. Differences in the changes from before ingestion in serum uric acid and triglyceride concentrations between active vs. placebo-treated subjects were analyzed using a sign test, Wilcoxson signed-rank test, or Mann–Whitney *U* test, and the data are presented as medians with interquartile ranges. Differences were considered significant when the probability of no difference was less than 5%.

## 3. Results

From 178 recruited subjects, only 32 healthy subjects aged 21–62 years (47.9 ± 9.2) with mild hyperuricemia were randomly assigned to groups 1 or 2. One subject (No.28) was assigned to Group 2 and dropped out during period 1 in the crossover study due to concerns about chronic prostatitis. Therefore, final analyses were performed using data from 31 subjects (male, 30; female, 1). Rates of medication compliance, which was managed through the daily records of each subject, were 99.5% in period 1 and 99.7% in period 2. The characteristics of the subjects were shown in Table 2.

Changes in serum uric acid concentrations were the main outcome variable during the trial, and these are summarized in Table 3. Individual data of the subjects was shown in Table S1 as Supplementary Material. In the present crossover trial, neither the carryover effect (*p* = 0.911) nor the period effect (*p* = 0.748) were significant for serum uric acid levels. Serum uric acid levels after 6-week treatments (6.7 mg/dL) were significantly lower than at baseline (7.1 mg/dL) in the active treatment group, and no changes in uric acid levels were observed in the placebo group (7.0 mg/dL before vs. 6.9 mg/dL after ingestion). These changes differed significantly between active (−0.4 mg/dL) and placebo-treated subjects (−0.1 mg/dL). Additionally, stratified analyses were conducted using the definition of hyperuricemia [2], and similar results were obtained in both cases (> 7.0 and ≤ 7.0 mg/dL). Serum uric acid levels 6 weeks after ingestion were significantly lower than that before ingestion in the active treatment group, and no changes in serum uric acid levels were observed in the placebo group. Moreover, these changes indicated a significant difference between the active and the placebo-treated groups in both cases.

One hour urinary uric acid excretion, urate and creatinine clearances, urinary pH, and serum glycine and tryptophan concentrations are presented in Table 4. No changes in urinary uric acid contents or urate and creatinine clearance were observed before and after ingestion in either active or placebo-treated subjects. After excluding one subject who exceeded the normal range at week 0, urate clearance in the active (*n* = 30) group was significantly increased (*p* = 0.027). No changes in urate clearance rates were observed in the placebo group. Similarly, urinary pH increased significantly after ingestion of amino acids, whereas no significant changes were observed in the placebo group. Serum glycine concentrations were increased after administration of amino acids and no such changes were seen in the placebo group. Both active and placebo groups had stable serum tryptophan levels before ingestion and after 6 weeks.

Multiple additional analyses were conducted to explore unknown physiological effects, and to confirm the safety of continuous ingestion of glycine and tryptophan mixtures for 6 weeks (Table 5 and Table 6). These analyses showed that platelet counts, serum creatinine and HDL-C levels changed significantly in the active treatment group. In addition, serum albumin and HDL-C levels changed significantly after ingestion of placebo. Serum triglyceride levels were also decreased significantly after ingestion of the active powder. In the present crossover trial, the carryover effect (*p* = 0.021) was significant for serum triglyceride levels. Thus, detailed analyses were performed using the data during the Period 1 (0–6 weeks) as shown in Table 6. Serum triglyceride levels 6 weeks later were significantly lower than before ingestion in Group 1 (active treatment group). The changes from baseline were significantly lower in Group 1 than in Group 2 (placebo group). Moreover, stratified analyses were performed using the diagnostic criteria of hypertriglyceridemia [17]. We performed further analyses of serum triglyceride levels in subjects with levels of under 150 mg/dL and not less than 150 mg/dL before the trial. In these analyses, ingestion of the active mixture in Group 1 significantly decreased serum triglyceride levels compared with those in Group 2 in under 150 mg/dL. Statistical analysis was not performed for the subjects with hypertriglyceridemia (not less than 150 mg/dL) because the small sample size (*n* = 3).

## 4. Discussion

Before planning this clinical trial, an animal study was conducted to identify amino acids that synergize with glycine to produce effective future functional foods for human consumption. In this study, combined doses of 2.0 g/kg glycine and 0.133 g/kg tryptophan significantly increased urinary urate excretion from Wistar rats after 7 h. In these experiments, cumulative uric acid excretion was 0.97, 1.07, 0.88, and 1.22 mg at 7 h after treatment with water, 2.0 g/kg glycine, 0.133 g/kg tryptophan, and 2.0 g/kg glycine with 0.133 g/kg tryptophan, respectively (unpublished data). Later, we confirmed that single combination doses of 3–6 g glycine with small quantities (0.1–0.4 g) of tryptophan transiently lowered serum uric acid more effectively than doses of glycine alone in a preliminary clinical study. The daily combined dosage was settled as an appropriate amount in continual supplementation.

Herein, we demonstrated that daily ingestion of 3.0 g of glycine with 0.2 g of tryptophan for 6 weeks decreases serum uric acid concentrations in subjects with mild hyperuricemia (serum uric acid, 6.6–7.9 mg/dL). Glycine is likely to increase urinary excretion of uric acid in rats and in healthy male humans [10,11]. Yet little is known about uric acid-lowering effects of tryptophan. It is clear from the present data that the mixture of glycine and tryptophan enhanced urate clearance in the cases, except for one wherein the subject had abnormally high urate clearance. Maximum serum levels of glycine or tryptophan would be achieved within an hour of ingestion, and would be followed by rapid decreases as the amino acids are metabolized. When we measured urinary uric acid levels, no changes followed ingestion of the active amino acid mixture, likely because our fasting measurements of urine parameters were taken after the changed serum and urine metabolite concentrations had returned to normal levels. The solubility of uric acid increases with an increase in pH [18]. Alternatively, glycine has a buffering effect and is often used as an anti-acid agent. Potentially, increases pH due to daily ingestion of glycine and tryptophan for 6 weeks enhanced the solubility of urinary uric acid, and thus elevated urate clearance. Other mechanisms are also conceivable, and pyrazinamide, which is used to treat tuberculosis, reportedly disabled glycine-induced uricosuria [19]. These effects of pyrazinamide may reflect enhanced urate reabsorption following exchange of its active metabolite [20]. The urate transporter 1 (URAT1) is the primary re-absorptive urate transporter that is targeted by pyrazinamide [21]. Thus, our results may reflect inhibition of the re-absorptive action of uric acid by URAT1. In this regard, it is inferred that tryptophan promote direct action of glycine against URAT1 because behavior of tryptophan to URAT1 is structurally difficult. Nonetheless, we suggest that, under the present conditions, increased uric acid excretion is primarily due to synergistic effects of glycine and tryptophan. Further studies are required to clarify the uric acid-lowering mechanisms of glycine and tryptophan. 

In further experiments, the combined treatments with glycine and tryptophan decreased serum triglyceride levels, and this lowering effect was also observed in subjects with triglyceride level under 150 mg/dL. The supplementation of the amino acid mixture was likely to decrease serum triglyceride level in hypertriglyceridemia although the effect was uncertain due to few subjects. Both moderate and severe hypertriglyceridemia commonly reduces HDL levels and increases levels of atherogenic small dense LDL, and are associated with substantially increased long term total mortality and cardiovascular risk [22]. To our knowledge, no previous reports show positive effects of glycine on serum triglyceride levels. In another study, however, supplementation with 15 g of glycine per day for three months did not affect serum triglyceride concentrations in patients with metabolic syndrome [23]. In contrast, treatment with 1.0 g of L-tryptophan or 0.01 g of melatonin, a metabolite of tryptophan, for 6 weeks or 14 months reportedly decreased serum triglyceride levels and reduced concentrations of pro-inflammatory cytokines such as interleukin-1, -6, and tumor necrosis factor-alpha in patients with steatohepatitis or nonalcoholic fatty liver disease [24,25]. Hence, perhaps the triglyceride-lowering effects of mixtures of glycine and tryptophan are mediated by melatonin, which is a metabolite of tryptophan. 

The safety of glycine and tryptophan treatments can be assumed because these amino acids are present in proteins and dietary supplements that are ingested in normal daily lives. Moreover, up to 90 g of glycine per day was administered over several weeks without serious adverse effects in clinical trials [26]. Similarly, oral administration of up to 5.0 g of L-tryptophan per day did not cause any adverse effects in young adult females [27]. During this clinical trial, no adverse effects were attributed to mixed glycine and tryptophan supplements. However, platelet counts and serum creatinine levels were significantly decreased by the present amino acid treatments, although these changes did not exceed normal values in any of our subjects. Collectively, this and other studies confirm the safety of oral treatments with the mixture of 3.0 g of glycine and 0.2 g of tryptophan. 

With regard to the limitations of this study, the administration period of crossover regimens was only 6 weeks. Hence, longer trials may show greater benefits of these amino acids in subjects with mild hyperuricemia, and such studies would be best performed as parallel-group comparisons. Furthermore, male to female ratio indicated a disproportionate number. Further research might be needed, although our preliminary study had suggested that there was no effective difference between males and females by the amino acids supplementation.

## 5. Conclusions

Combined daily supplementation with 3.0 g of glycine and 0.2 g of tryptophan for 6 weeks significantly decreased serum uric acid concentrations in 31 subjects (male, 30; female, 1) with mild hyperuricemia. The associated increases in solubility of uric acid caused by the elevation of urinary pH are likely related to lowered serum uric acid levels. In addition, combined amino acid treatments significantly decreased serum triglyceride concentrations in all subjects and in those with under 150 mg/dL. In future studies, the therapeutic and preventive effects of this mixture of amino acids may be described in more detail.

## Figures and Tables

**Table 1:** Components of the test foods

Components	Active (g)	Placebo (g)
Glycine	3.0	-
L-Tryptophan	0.2	-
Dextrin	2.0	5.2

**Table 2:** Characteristics of the subjects.

	Group 1 (Active then Placebo)	Group 2 (Placebo then Active)	Total Subjects	Complete Subjects for Analyses
	*n* = 16	*n* = 16	*n* = 32	*n* = 31
Age	47.9 (9.2)	47.8 (9.4)	47.9 (9.2)	47.9 (9.3)
Sex				
Male	16	15	31	30
Female	0	1	1	1
BMI	24.2 (2.4)	23.9 (3.9)	24.1 (3.2)	24.1 (3.2)

Data are presented as means (standard deviations).

**Table 3:** Summary of serum uric acids concentrations (mg/dL) in the crossover trial.

	Period 1			Period 2		Change in Period 1	Change in Period 2
	0w	6w		10w	16w	(6w–0w)	(16w–10w)
Group 1 (Active then Placebo), *n* = 16	7.1 (0.7)	6.7 (0.5)		6.9 (0.6)	6.9 (0.5)	0.4 (0.7)	−0.1 (0.5)
Group 2 (Placebo then Active), *n* = 15	7.0 (0.6)	6.9 (0.6)		7.1 (0.7)	6.6 (0.7)	−0.1 (0.7)	−0.4 (0.8)
	Before Ingestion		6 Weeks Later	*p* Value ^a^		Change from Before Ingestion	*p* Value ^b^
Active, *n* = 31	7.1 (0.7)		6.7 (0.6)	0.004		−0.4 (1.1)	0.028
Placebo, *n* = 31	7.0 (0.6)		6.9 (0.5)	0.526		−0.1 (0.8)	
Stratified analyses: the serum uric acid levels of 7.0 mg/dL at the screening before the trial more than 7.0 mg/dL							
Active, *n* = 17	7.3 (0.8)		6.8 (0.6)	0.042		−0.5 (1.1)	0.015
Placebo, *n* = 17	7.3 (0.6)		7.0 (0.5)	0.051		−0.3 (0.7)	
7.0 mg/dL or less							
Active, *n* = 14	6.8 (0.4)		6.5 (0.5)	0.042		−0.4 (0.9)	0.003
Placebo, *n* = 14	6.6 (0.4)		6.8 (0.6)	0.183		0.2 (0.7)	

Data are presented as means (standard deviations); ^a^ paired *t*-test between before ingestion and 6 weeks later; ^b^ sign test (*n* = 31) or the Wilcoxson signed-rank test (stratified analyses) of changes from before ingestion in active and placebo groups. These nonparametric data are expressed as median (interquartile ranges). Differences were considered significant when *p* < 0.05. Stratified analyses were performed in subjects using the definition of hyperuricemia (serum uric acid levels: 7.0 mg/dL) at baseline.

**Table 4:** Changes in urine parameters and serum amino acid levels in active and placebo groups.

Parameters	Groups	Before Ingestion	6 Weeks Later	*p* Value ^a^
Urinary uric acid (mg/kg/h)	Active, *n* = 31	0.434 (0.178)	0.432 (0.157)	0.928
	Placebo, *n* = 31	0.414 (0.122)	0.437 (0.178)	0.373
Urate clearance (mL/min)	Active, *n* = 31	6.8 (2.7)	7.3 (3.0)	0.125
	Placebo, *n* = 31	6.6 (1.9)	7.1 (3.2)	0.232
Exclusion of the one subject *				
	Active, *n* = 30	6.6 (2.5)	7.3 (3.0)	0.027
	Placebo, *n* = 30	6.6 (1.9)	7.1 (3.2)	0.302
Creatinine clearance (mL/min)	Active, *n* = 31	131 (36)	133 (37)	0.683
	Placebo, *n* = 31	129 (26)	138 (68)	0.341
Urinary pH	Active, *n* = 31	5.7 (0.6)	6.0 (0.6)	0.005
	Placebo, *n* = 31	5.7 (0.5)	5.9 (0.8)	0.196
Glycine (nmol/mL)	Active, *n* = 31	124 (29)	141 (33)	0.014
	Placebo, *n* = 31	126 (23)	128 (28)	0.661
L-Tryptophan (nmol/mL)	Active, *n* = 31	52 (10)	53 (8)	0.748
	Placebo, *n* = 31	50 (8)	52 (7)	0.185

Data are presented as means (standard deviations); ^a^ paired *t*-test comparing levels before ingestion with those recorded 6 weeks later. Differences were considered significant when *p* < 0.05. Urinary uric acid contents and urate and creatinine clearance rates were analyzed and calculated in urine specimens that were collected for 60 min after complete urine excretion following ingestion of 500 mL of water; * Urate clearance in subject No.18 at 0 weeks exceeded the maximum limit of the normal range (6.2–12.6). Abbreviation: potential of hydrogen (pH).

**Table 5:** Changes in clinical serum parameters in active and placebo groups.

Parameters	Groups	Before Ingestion	6 Weeks Later	*p* Value ^a^	Parameters	Groups	Before Ingestion	6 Weeks Later	*p* Value ^a^
Body weight (kg)	Active, *n* = 31	69.4 (9.4)	69.7 (9.0)	0.209	AST	Active, *n* = 31	20 (6)	21 (5)	0.645
	Placebo, *n* = 31	69.4 (9.4)	69.5 (9.6)	0.388	(U/L)	Placebo, *n* = 31	20 (5)	21 (6)	0.179
Body mass index (kg/m^2^)	Active, *n* = 31	24.1 (3.3)	24.2 (3.2)	0.202	ALT	Active, *n* = 31	21 (13)	21 (11)	0.845
	Placebo, *n* = 31	24.1 (3.2)	24.2 (3.4)	0.231	(U/L)	Placebo, *n* = 31	22 (12)	21 (9)	0.593
Systolic blood pressure (mmHg)	Active, *n* = 31	121 (9)	123 (8)	0.241	LDH	Active, *n* = 31	185 (59)	182 (28)	0.696
	Placebo, *n* = 31	123 (10)	124 (9)	0.739	(U/L)	Placebo, *n* = 31	178 (35)	179 (31)	0.810
Diastolic blood pressure (mmHg)	Active, *n* = 31	74 (6)	76 (6)	0.338	GGT	Active, *n* = 31	36 (23)	36 (22)	0.891
	Placebo, *n* = 31	75 (7)	76 (7)	0.333	(U/L)	Placebo, *n* = 31	35 (22)	37 (25)	0.136
White blood cell counts (/μL)	Active, *n* = 31	5626 (1181)	5858 (1495)	0.339	ALP	Active, *n* = 31	185 (45)	184 (47)	0.634
	Placebo, *n* = 31	5845 (1198)	5706 (1327)	0.387	(U/L)	Placebo, *n* = 31	197 (60)	188 (44)	0.331
Red blood cell counts (10^4^/μL)	Active, *n* = 31	494 (34)	489 (36)	0.133	Total bilirubin	Active, *n* = 31	0.9 (0.4)	0.8 (0.3)	0.752
	Placebo, *n* = 31	491 (35)	494 (36)	0.306	(mg/dL)	Placebo, *n* = 31	0.8 (0.3)	0.8 (0.3)	0.193
Hemoglobin (g/dL)	Active, *n* = 31	15.1 (1.1)	14.9 (1.1)	0.095	Creatinine	Active, *n* = 31	0.85 (0.14)	0.83 (0.12)	0.039
	Placebo, *n* = 31	15.0 (1.0)	15.0 (1.1)	0.526	(mg/dL)	Placebo, *n* = 31	0.85 (0.12)	0.85 (0.12)	0.465
Hematocrit (%)	Active, *n* = 31	45.4 (2.6)	45.2 (2.9)	0.605	BUN	Active, *n* = 31	13.6 (4.3)	13.5 (3.7)	0.756
	Placebo, *n* = 31	45.2 (2.8)	45.6 (3.0)	0.066	(mg/dL)	Placebo, *n* = 31	13.3 (3.1)	13.4 (5.0)	0.828
Platelet counts (10^4^/μL)	Active, *n* = 31	26.0 (4.7)	25.2 (4.8)	0.021	HBA1c	Active, *n* = 31	5.4 (0.3)	5.4 (0.3)	0.647
	Placebo, *n* = 31	26.6 (5.3)	25.4 (4.9)	0.059	(%)	Placebo, *n* = 31	5.4 (0.3)	5.3 (0.3)	0.608
Total protein (g/dL)	Active, *n* = 31	7.2 (0.5)	7.2 (0.5)	0.659	HDL-C	Active, *n* = 31	60 (12)	62 (13)	0.049
	Placebo, *n* = 31	7.1(0.5)	7.2 (0.5)	0.077	(mg/dL)	Placebo, *n* = 31	58 (13)	61 (14)	0.012
Albumin (g/dL)	Active, *n* = 31	4.4 (0.3)	4.3 (0.3)	0.276	LDL-C	Active, *n* = 31	120 (30)	118 (33)	0.619
	Placebo, *n* = 31	4.3 (0.3)	4.4 (0.3)	0.007	(mg/dL)	Placebo, *n* = 31	114 (27)	119 (34)	0.190
Glucose (mg/dL)	Active, *n* = 31	88 (6)	90 (8)	0.321	Triglyceride	Active, *n* = 31	113 (49)	97 (52)	0.031
	Placebo, *n* = 31	88 (6)	88 (7)	0.494	(mg/dL)	Placebo, *n* = 31	109 (60)	113 (68)	0.692

Data are presented as means ± standard deviations; ^a^ paired t-tests were used to compare values between before ingestion and 6 weeks later. Differences were considered significant when *p* < 0.05. Abbreviations: aspartic aminotransferase (AST), alanine aminotransferase (ALT), lactate dehydrogenase (LDH), gamma-glutamyl transferase (GGT), alkaline phosphatase (ALP), blood urea nitrogen (BUN), HDL-cholesterol (HDL-C), LDL-cholesterol (LDL-C), and hemoglobin A1c (HBA1c).

**Table 6:** Comparison of serum triglyceride concentrations (mg/dL) in active and placebo groups.

	Period 1		*p* Value ^a^	Change from Before Ingestion	*p* Value ^b^
	0w	6w			
Group 1 (Active), *n* = 16	119 (48)	86 (34)	0.002	−34 (44)	0.004
Group 2 (Placebo), *n* = 15	109 (72)	129 (89)	0.310	6 (39)	
Stratified analyses: the serum triglyceride levels of 150 mg/dL at the screening before the trial					
<150 mg/dL					
Group 1 (Active), *n* = 13	106 (36)	78 (22)	0.001	−31 (30)	0.003
Group 2 (Placebo), *n* = 12	86 (36)	113 (91)	0.219	5 (26)	
not less than 150 mg/dL					
Group 1 (Active), *n* = 3	174 (64)	120 (61)	-	−60 (70)	-
Group 2 (Placebo), *n* = 3	200 (118)	194 (43)	-	33 (89)	

Data are presented as means (standard deviations); ^a^ paired t-tests were used to compare triglyceride levels before ingestion and 6 weeks later; ^b^ Mann–Whitney U tests were performed for the comparison of changes from before ingestion in active and placebo groups. Data are expressed as medians (interquartile ranges) as nonparametric data. A *p* value of < 0.05 was considered to be statistically significant. Stratified analyses were performed in subjects with levels of under 150 mg/dL or not less than 150 mg/dL at baseline. Statistical analysis was not performed for subjects with hypertriglyceridemia (not less than 150 mg/dL) because of the small sample size (*n* = 3).

## Supplementary Materials

The following are available online at https://www.mdpi.com/2072-6643/11/3/564/s1. Table S1: Individual data (*n* = 31) of serum uric acids concentrations (mg/dL) in the crossover trial.
